# Supplementary material for: The RNA-binding profile of the splicing factor SRSF6 in immortalized human pancreatic β-cells
Source: Life Sci Alliance. 2020 Dec 29;4(3):e202000825. doi: 10.26508/lsa.202000825 (PMC7772782; doi:10.26508/lsa.202000825)
Supplement: Supplementary file 9 [file LSA-2020-00825_TableS3.docx]

**Supplementary Table S3. Sequence of the antisense oligonucleotides (AONs).**

| **ASO** | **Supplier** | **Sequence (5' - 3')** | **Structural modifications** |
| --- | --- | --- | --- |
| ASO-3ss  (targeting 3’ splice site of *LMO7* exon 10) | Eurogentec, Liége, Belgium | GAAAGAGUCUAAGAAACC | 2’-*O*-methyl RNA nucleosides and a full-length phosphorothioate backbone |
| ASO-5ss (targeting 5’ splice site of *LMO7* exon 10) | Eurogentec, Liége, Belgium | CACACAACUUACCCAUUC | 2’-*O*-methyl RNA nucleosides and a full-length phosphorothioate backbone |
| ASO-Ctrl (control) | Eurogentec, Liége, Belgium | CTCATTCCTACCGACACCCC | 2’-*O*-methyl RNA nucleosides and a full-length phosphorothioate backbone |
