# Supplementary material for: The RNA-binding profile of the splicing factor SRSF6 in immortalized human pancreatic β-cells
Source: Life Sci Alliance. 2020 Dec 29;4(3):e202000825. doi: 10.26508/lsa.202000825 (PMC7772782; doi:10.26508/lsa.202000825)
Supplement: Supplementary file 10 [file LSA-2020-00825_TableS4.docx]

**Supplementary Table S4. List of siRNAs used.**

| **Name** | **Supplier** | **Sequence (5' - 3')** |
| --- | --- | --- |
| siCTL (Allstars Negative Control siRNA) | Qiagen, Venlo, Netherlands | Not provided |
| siSRSF6#2 Silencer Select siRNAi | Invitrogen, Pasley, UK | CCUGUUCGUACAGAAUACAGGCUUA |
| siGLIS3 Silencer Select siRNAi | Invitrogen, Pasley, UK | CAGCAAUAGUGUCUCUAACUCAUUA |
