# Supplementary material for: The RNA-binding profile of the splicing factor SRSF6 in immortalized human pancreatic β-cells
Source: Life Sci Alliance. 2020 Dec 29;4(3):e202000825. doi: 10.26508/lsa.202000825 (PMC7772782; doi:10.26508/lsa.202000825)
Supplement: Supplementary file 11 [file LSA-2020-00825_TableS5.docx]

**Supplementary Table S5. List of primary and secondary antibodies used.**

| **Antibody** | **Supplier** | **Identifier** | **Dilution** |
| --- | --- | --- | --- |
| SRSF6/SRp55 (rabbit) | LifeSpan Bioscience | LS-C290327 | 1:1000 |
| α-Tubulin (mouse) | Sigma Aldrich | T5168 | 1:5000 |
| Peroxidase- conjugated donkey anti-rabbit IgG | Jackson ImmunoResearch | Cat#715-036-152; RRID:AB_2340590 | 1:5000 |
|  |  |  |  |
| Peroxidase- conjugated donkey anti-mouse IgG | Jackson ImmunoResearch | Cat#711-036-150; RRID:AB_2340773 | 1:5000 |
|  |  |  |  |
