# Supplementary material for: The RNA-binding profile of the splicing factor SRSF6 in immortalized human pancreatic β-cells
Source: Life Sci Alliance. 2020 Dec 29;4(3):e202000825. doi: 10.26508/lsa.202000825 (PMC7772782; doi:10.26508/lsa.202000825)
Supplement: Supplementary file 12 [file LSA-2020-00825_TableS6.docx]

**Supplementary Table S6. Summary of SRSF6 iCLIP experiments in EndoC-βH1 cells.** The iCLIP experiment was performed in four independent replicates, which yielded a total of 68,449,054 crosslink events.

| **Replicate** | **Sample ID** | **Barcode** | **Sequenced reads** | **Crosslink events** |
| --- | --- | --- | --- | --- |
| Rep 1 | imb_koenig_2017_10_JKRS33_SRSF6_rep1 | NNNCGCCNN | 29,496,620 | 12,318,770 |
| Rep 2 | imb_koenig_2017_10_JKRS33_SRSF6_rep2 | NNNTACGNN | 77,766,901 | 27,542,289 |
| Rep 3 | imb_koenig_2017_10_JKRS33_SRSF6_rep3 | NNNATACNN | 38,186,626 | 16,161,814 |
| Rep 4 | imb_koenig_2017_10_JKRS33_SRSF6_rep4 | NNNCGAGNN | 29,936,638 | 12,426,181 |
