# Supplementary material for: The RNA-binding profile of the splicing factor SRSF6 in immortalized human pancreatic β-cells
Source: Life Sci Alliance. 2020 Dec 29;4(3):e202000825. doi: 10.26508/lsa.202000825 (PMC7772782; doi:10.26508/lsa.202000825)
Supplement: Supplementary file 13 [file LSA-2020-00825_TableS7.docx]

**Supplementary Table S7. Sequences of primers used for splicing analyses and quantitative RT-PCR**. Abbreviations used are as follows: SPL, primers used to analyze splicing variants; qRT, primers used for quantitative RT-PCR.

| **Gene** | **Application** | **Forward (5'-3')** | **Reverse (5'-3')** |
| --- | --- | --- | --- |
| *LMO7* | SPL | CCTATGTACCAGCACCTCTG | CCATTTTGCAAGGTCATCCTGC |
| *RBM6* | SPL | GGTACCTGAAGATGCCACAAAAG | CCACCAATGTTTGCCTTACATCG |
| *ITGB3BP* | SPL | GCCTGTTAAAAGATCACTGAAG | CTACTGCCCTCCAAAGCCTGTAT |
| *STARD10* | SPL | CCCTGAAGAACCGTGATGTC | CTTCTTCATGGCCTTGGGAGC |
| *CDK2* | SPL | CATCAAGAGCTATCTGTTCCAGC | GCATAGAAGTAACTCCTGGCC |
| *CENPO* | SPL | GGGAATTCTCGCTTCTGGCCTG | GTTCCAAGAGCACCTTCCTGGG |
| *BCAR1* | SPL | CAAAGGTGGTGGTGCCCACC | CACGTCGTAGAGGTCAGGAGCC |
| *ACTB* | qRT | CTGTACGCCAACACAGTGCT | GCTCAGGAGGAGCAATGATC |
| *SRSF6 (SRp55)* | qRT | CATAGGACGCCTGAGCTACA | TGCCGTTCAGCTCGTAAAC |
| *GLIS3* | qRTPCR | CAACCAGATCAGTCCTAGCTTACA | GCGAAATAAGGGACCTGGTATC |
